# Total Hip Arthroplasty in a Patient with Mucopolysaccharidosis Type IVB

**DOI:** 10.1155/2021/5584408

**Published:** 2021-04-28

**Authors:** Yannick N. T. van den Eeden, Niklas Unter Ecker, Holger Kleinertz, Thorsten Gehrke, Tobias M. Ballhause

**Affiliations:** Department of Orthopedic Surgery, ENDO-Klinik Hamburg, Holstenstr. 2, 22767 Hamburg, Germany

## Abstract

*Introduction*. Morquio syndrome or mucopolysaccharidosis (MPS) type IV is a rare autosomal recessive lysosomal storage disease, characterized by abnormal metabolism of glycosaminoglycans associated with specific skeletal deformities, also known as dysostosis multiplex. *Case Presentation*. We present the case of a 23-year-old patient with advanced osteonecrosis of the femoral head (ONFH) on both sides due to Morquio syndrome. A diagnosis of mucopolysaccharidosis type IVB was made after extensive genetic profiling. The patient had the condition for a long time. At 7 years old, the patient was treated with bilateral pelvic Salter's osteotomy. Afterward, the patient was able to walk freely but could never take part in sports. At 22 years old, pain in the hip increased, and magnetic resonance imaging showed a bilateral femur head necrosis. Hence, the patient underwent cementless total hip arthroplasty (THA). Intraoperatively, a periprosthetic fracture occurred. Therefore, revision surgery with internal fixation was performed on the next day. Postoperatively, a weight-bearing restriction of 20 kg on the left leg was imposed for 6 weeks. The patient made a full recovery and was able to move without residual complaints. Annual orthopedic evaluation in patients treated with surgical intervention is recommended. *Discussion*. Orthopedic challenges for mucopolysaccharidoses and corresponding bone alterations, known as dysostosis multiplex, involving trunk and limbs with typical radiological findings have been well described. The hip is invariably involved, with dysplasia affecting the femoral neck (coxa valga), femoral epiphysis (loss of sphericity, osteonecrosis), and a flared hypoplastic iliac wing. Symptomatic therapy consists, on the one hand, of a surgical procedure and, on the other hand, a variety of supportive measures. However, the management of joint replacement in lysosomal storage diseases has not been well reported. All patients with MPS should be considered at high risk for surgical intervention requiring anesthesia because of airway and cardiac disease manifestations. In the case of a need for THA, we recommend cemented stem fixation because of the overall poor bone quality in patients with Morquio syndrome.

## 1. Introduction

Mucopolysaccharidoses (MPS) are a group of rare, inherited lysosomal storage disorders. A genetic defect causes a disruption in the breakdown of complex carbohydrates, also known as glycosaminoglycans (GAG). Nondegradable substrates accumulate in the lysosomes and cause dysfunctions in various organ-systems [1]. The resulting disturbances of cell metabolism lead to progressive, multiorgan damage and increased mortality, with varying clinical manifestations depending on the type of accumulated substrate [2]. According to clinical and biochemical characteristics, seven distinct clinical types of MPS (MPS I, II, III, IV, VI, VII, and IX) can be distinguished, which in turn can be divided into different subtypes [1, 3]. The distinct types of MPS also have names (in addition to the Roman numerals), e.g., Scheie's syndrome (MPS I) or Hunter's disease (MPS II). Although an individual MPS is rare, MPS are relatively frequent as a group, with an overall incidence estimated as 1 : 22,000 [1]. With the exception of MPS II, all MPS forms have an autosomal recessive inheritance. Patients typically appear normal at birth, but they experience the onset of clinical disease during early childhood [3]. The most frequent form of MPS is type II, which accounts for 29% of all MPS [4]. Osteoarticular abnormalities, including anomalies of the skull, spinal deformations, and development dysplasia of the hip and genu valgum, are pathognomonic for all forms of MPS [5]. Radiologically, the skeletal deformities are labeled as dysostosis multiplex, characterized by severe abnormalities in the development of skeletal cartilage and bone [5].

MPS type IV (Morquio syndrome) is characterized by spondylo-epiphyseo-metaphyseal dysplasia. There are two subtypes, A and B, depending on the deficiency of one of two enzymes involved in the breakdown of keratin sulfate (KS) [6, 7], N-acetylgalactosamine-6-sulfate sulfatase in MPS IVA and beta-D-galactosidase in MPS IVB [8, 9]. The incidence ranges between 1 : 76,000 and 1 : 450,000 births, with great differences among individual countries [10]. Altogether, MPS IV accounts for 24% of all MPS [4]. Clinically, the two subtypes cannot be distinguished because they both occur with different degrees of severity, but patients with MPS IVB typically have a milder disease course than patients with MPS IVA. The accumulation of KS in cartilage, as opposed to bone, is responsible for the skeletal manifestations characteristic of MPS IV types A and B [11]. In addition to bone and cartilage lesions, patients with MPS type IV have respiratory problems due to tracheal obstruction and heart valve disease as well as cervical spinal cord complications [12]. The prognosis is determined by the severity of the disease and the quality of treatment. With good treatment, patients with Morquio syndrome can live beyond 50 years old [13].

The hip is invariably involved, with development dysplasia affecting the femoral neck (coxa valga), the femoral epiphysis (loss of sphericity, osteonecrosis), and the femoral acetabulum [14]. Changes in hip morphology may contribute to hip instability with a tendency to lateral and proximal migration. Therefore, surgical hip reconstruction for safe containment of the femoral head is frequently necessary during infancy [5, 14]. However, once the cartilage is damaged to a large extent, total hip arthroplasty (THA) is the only rational therapeutic option [15].

Here, we describe THA in a patient with advanced ONFH on both sides due to genetically confirmed MPS IV type B. Furthermore, possible complications due to reduced bone quality are discussed.

## 2. Case Presentation

We report a 23-year-old Caucasian male patient with advanced femoral head necrosis on both sides. The patient had mucopolysaccharidosis type IVB. The mucopolysaccharidosis was only diagnosed specifically in 2018 by genetic examination. In line with the ONFH, the patient has osteonecrosis of the left upper ankle joint. Typically, for mucopolysaccharidosis, the vertebral column shows cervicothoracic kyphoscoliosis.

**Figure 1:**
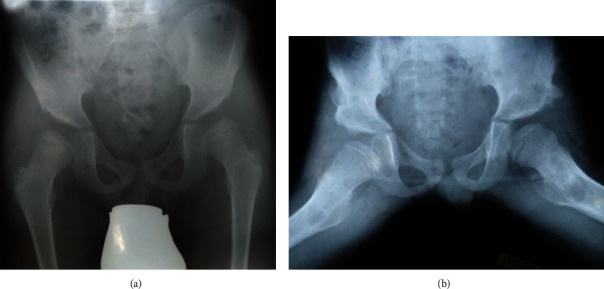
Severe hip dysplasia. Preoperative X-ray images of the patient at 7 years old. Image (a) depicts the pelvis on an anteroposterior X-ray projection. The acetabular angle (AC angle) is 26° and 35° on the left and right, respectively. The lateral center-edge angle (LCE angle) is 14° on the left hip and 8° on the right. Image (b) shows Rippstein's projection of the patient's pelvis.

**Figure 2:**
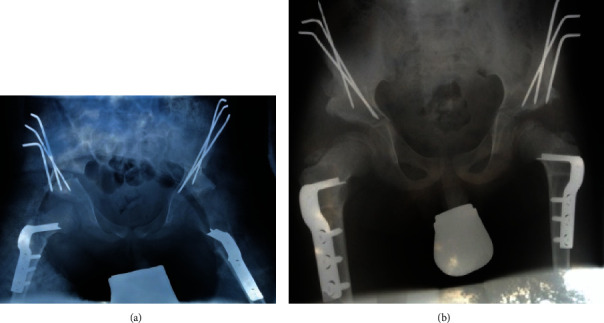
Postoperative result after Salter's triple osteotomy and VDRO on both sides. Image (a) shows the direct postoperative situation after surgery. Image (b) shows a consolidation of the osteotomies 6 weeks after surgery, prior to the removal of the k-wires.

**Figure 3:**
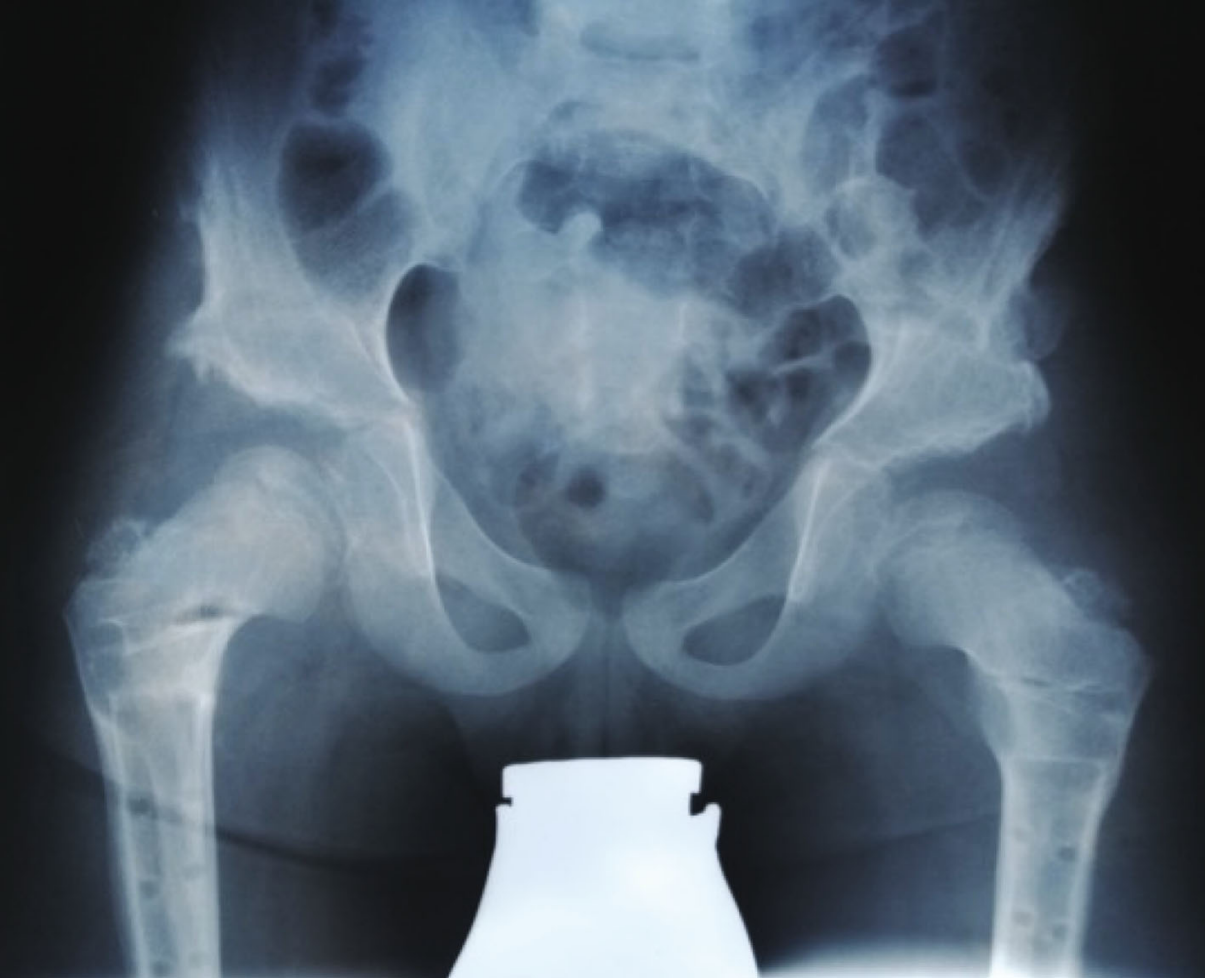
Result of the Salter osteotomy and VDRO at 8 years old. One year after bilateral surgery to remove the femoral implants. The AC angle is 16° on the left side and 20° on the right side. The LCE angle is 34° on the left hip and 30° on the right side.

The patient has been affected for a long time. The parents of our patient are both Caucasian, but they are not consanguineously related. The patient's birth and postpartum course were unremarkable. Motor and linguistic milestones were reached in accordance with age. At 6 years old, hip dysplasia was diagnosed on both sides, which was indicated by an increasingly abnormal gait pattern (Figure 1). At 7 years old, a Salter innominate osteotomy and a varus derotation osteotomy (VDRO) were performed on both sides with subsequent improvement of symptoms (Figure 2). Surgery was performed in the pediatric department of a university hospital. Prior to the operation, the lateral center-edge angle (LCE angle) was 14° on the left hip, and the acetabular angle (AC angle) was 26°. On the right side, the LCE was 8°, and the AC was 35°. Hip containment was significantly improved by the operation, which led to an LCE of 34° and an AC of 16° on the left hip. On the right hip, the LCE was 30°, and AC was 20° after surgery (Figure 3). The patient was unable to participate in high school sports.

The patient also described instability of the ligamentous apparatus, increased curvature of the spine, and pronounced pain in all joints, especially during physical activity and also at rest. He is 1.68 m tall and weighs 68 kg, with a body mass index (BMI) of 19.5. He is shorter compared to his two brothers, who are not affected. Because of myopia, he has been wearing glasses since he was 2 years old. Defecation, diuresis, and sleep are normal. He also has no recurrent infections or increased tooth enamel defects. His symptoms had always been classified as unclear myopathy and spondyloepiphyseal dysplasia of the hip until a genetic diagnosis was made, which pointed to MPS IVB. The primary disease did not affect mental abilities. The patient graduated from a regular high school.

**Figure 4:**
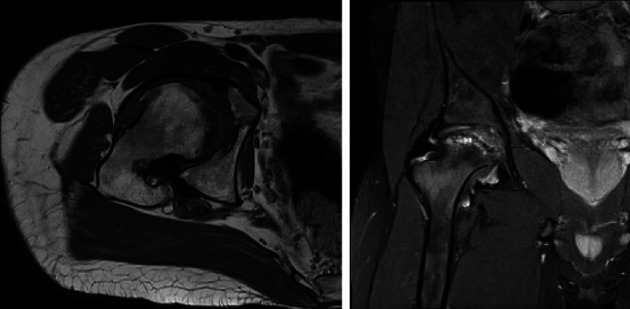
MRI of the right hip at the age of 22 years. The left image depicts the femoral head on the axial plane in a fat-saturated sequence. Edema can be observed in the femoral head. The right image shows the hip in coronal plane in a T1 sequence after application of contrast medium (gadolinium). The femur head has been deformed into a sickle-like shape.

**Figure 5:**
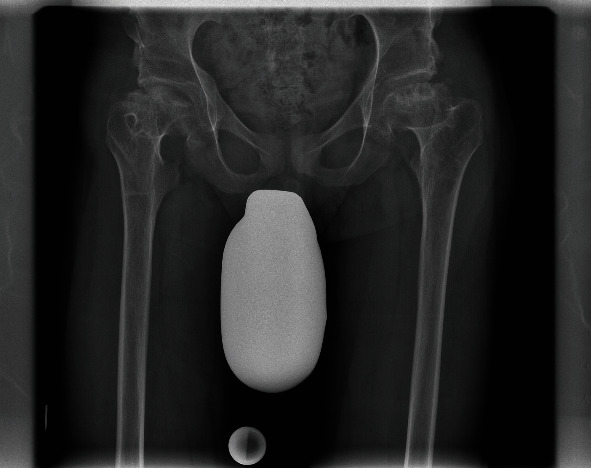
Advanced femoral head necrosis on both sides. The anteroposterior projection of the pelvis was made for planning the THA. Because of increasing pain on the right side, correlating with advanced femoral head necrosis, the patient requested THA surgery on the right hip.

Because of the increased hip pain, the patient consumed naproxen on a daily basis. The physician in charge ordered a magnetic resonance imaging (MRI) of the right hip when the patient was 22 years old. At that time, the right hip was more painful than the left. The MRI showed advanced ONFH (Figure 4). The patient contacted several physicians for their opinions regarding the treatment of advanced osteoarthritis of the hips. We recommended a cementless THA. At the time of the patient's introduction to our outpatient clinic, the left hip was more painful than the right. He stated that pain was 8 out of 10 on the numerical analog scale (NAS). The range of motion (ROM) in the hips was highly reduced. Hip extension/flexion according to the neutral-zero method was 5/0/95° on the right side and 10/0/100° on the left side. Abduction/adduction was 25/0/30° on the right side and 35/0/30° on the left side. Outward/inward rotation was 45/0/15° in the right hip and 40/0/30° in the left hip. An anteroposterior X-ray of the pelvis gave indicated advanced ONFH in both hips (Figure 5). Thus, we planned the arthroplasty on the left side.

**Figure 6:**
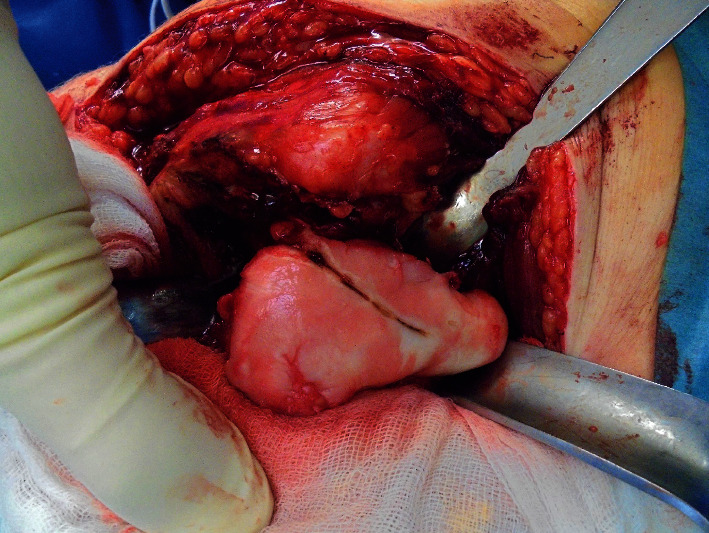
Intraoperative picture of the dysmorphic femoral head. The hip joint was approached from the posterior. After incision of the hip joint's capsule, the femoral head was luxated out of the acetabulum.

**Figure 7:**
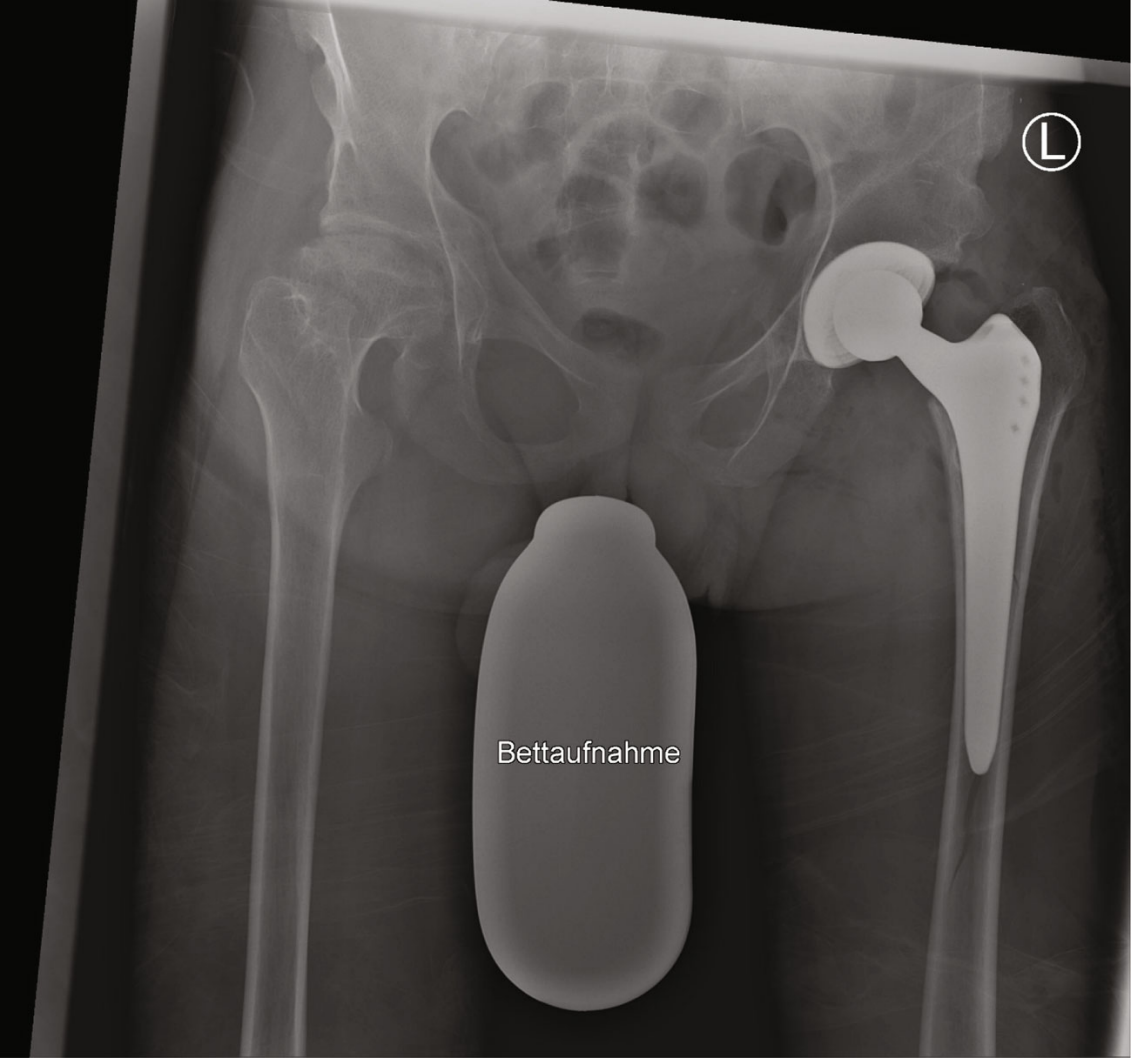
Postoperative image with periprosthetic fracture. This X-ray was taken in the postoperative care unit while the patient was lying in bed. A Vancouver type B1 fracture can be detected on the image. Hence, revision surgery was planned.

**Figure 8:**
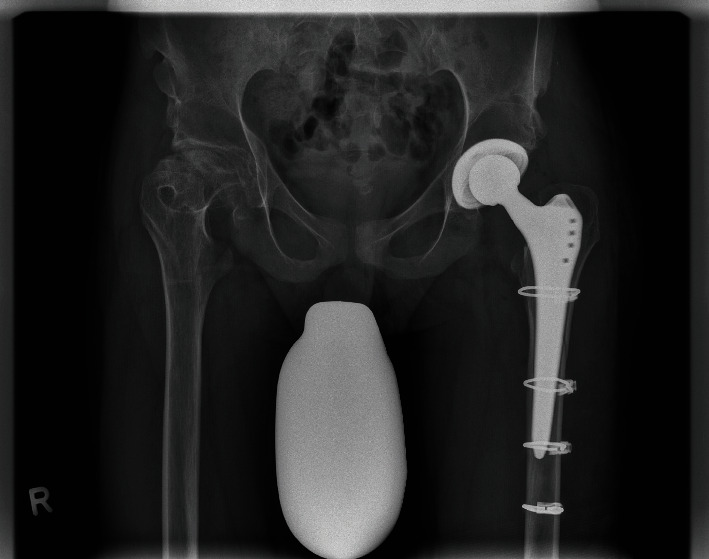
Osteosynthesis of the periprosthetic fracture. X-ray on the day of hospital discharge. The anteroposterior pelvic projections show the THA and osteosyntheses. The fracture has been reduced and fixed with four cable cerclages. The patient was restricted to 20 kg weight-bearing on the left leg for 6 weeks.

The patient was positioned on his right side on the operation table. The left leg was supported by a pillow to keep it in a neutral position. The hip was approached posteriorly [16]. Surgery was performed by a senior orthopedic surgeon with more than 10 years of experience in THA. During surgery, the whole dimension of ONFH became obvious (Figure 6). Implantation of the cup was not problematic (Allofit-Alloclassic Cup with Durasul-Inlay, Zimmer, Hamburg, Germany). During the hammering down process of the shaft component (Alloclassic SL-131°, size 3, Zimmer, Hamburg, Germany), the femoral shaft cracked. A Vancouver type B1 periprosthetic fracture was diagnosed (Figure 7). After a thorough explanation of the complication to the patient, revision surgery was undertaken on the next day. The femoral shaft was supported by four 1.8 mm cerclages (Cable-Ready, Zimmer*, Hamburg, Germany*) (Figure 8).

The patient was mobilized with physiotherapeutic assistance. A weight-bearing restriction of 20 kg on the left leg was imposed. The postoperative blood analysis showed normal values, and pain management was performed using metamizole (500 mg up to eight times per day) and oxycodone retard (10 mg two times per day for 3 days). Six days after the revision surgery, the patient was discharged. He undertook a rehabilitation program 4 weeks after surgery, which lasted for 3 weeks. The physical therapy protocol was tailored to the patient and included a variety of vocational and recreational activities.

## 3. Discussion

After an extensive search in the available literature, we found only a few existing case reports of unilateral and bilateral hip replacement in patients with mucopolysaccharidosis type IV [15, 17–19]. However, the confirmation of the diagnosis by enzyme assay or genetic profiling was generally not undertaken, and the condition was identified by its distinctive clinical features. Studies on the outcome of THA in patients with chondrodysplasias have been previously described [20–22]. In this case, we present a patient with genetically confirmed mucopolysaccharidosis type IV B with ONFH.

The parents of our patient are both Caucasian, but they are not consanguineously related. The skeletal deformities (platyspondylia, kyphosis, scoliosis, pectus carinatum, genu valgum, and deformities of the long bones) increase as the child grows but are usually not present until the first years of life. Hyperextensibility of the joints is accompanied by frequent luxation of the hip and knee joint. Skeletal involvement not only hinders walking and daily activities but also generally leads to arrested growth at about 8 years old, with a final height of 1–1.5 meters, depending on the severity of the disease. The skeletal deformities can also lead to neurological deficits. Atlantoaxial subluxation and spinal cord compression, especially in the upper cervical region, are common. Symptoms outside the skeleton include respiratory paralysis, hepatomegaly, cardiac abnormalities, hearing loss, and corneal opacity. Intelligence is normal [23].

Milder forms of these conditions with hip disorders have been successfully treated with bilateral varus osteotomies [24]. The regular hip containment is used when the LCE is 25 to 40°. An LCE below 20° is pathologic and characteristic of hip dysplasia. An LCE above 40° is an overcontainment of the hip, also known as Coxa profunda [25]. An AC of 16.8 to 19.3° has been reported to be physiologic in 7-year-old boys in Tönnis' original report on the AC [26]. No long-term results exist on the treatment of ONFH by drilling and muscle-pedicle bone grafting in patients with lysosomal storage diseases. In our case, advanced ONFH precluded both options. No reason for the development of ONFH can be directly correlated with MPS. ONFH might have been promoted by the altered hip morphology in this patient.

The treatment plan for MPS IV type A includes enzyme replacement therapy of the deficient enzyme [27]. Currently, there is no enzyme replacement therapy available for MPS IV type B [28]. In MPS VI, allogeneic bone marrow transplantation has no effect on skeletal symptoms. Therefore, the treatment is purely symptomatic and might include several surgeries throughout the patient's life.

The treatment plan for our patient was supportive treatment to improve quality of life, significantly improving physical capabilities while reducing the problems caused by the advanced femoral necrosis. With good treatment, patients with MPS IV can live to be over 50 years old. Because of the age of our patient, the chances of future revision surgery are high, considering potential linear abrasion and aseptic loosening of the implants [29, 30]. Therefore, an uncemented prosthesis was used with the idea to save bone stock and to simplify a possible revision in the future [31]. The specific model of uncemented femoral shaft was chosen in accordance with the radiological anatomy of the proximal femur (Alloclassic® Zweymueller® femoral hip stem). The use of a short stem was considered, but because of favoring proximal stress shielding and bone atrophy in the great trochanter and calcar regions, this was not used in our patient.

During the surgery, it was immediately clear that bone mineral density was low. Implantation of the acetabular component was uncomplicated. Additional influence on the overall architecture and bone quality of the proximal femur had osteosclerosis, which remained after VDRO. The proximal femur fractured after careful preparation and implantation of the shaft. In the postoperative X-ray, a Vancouver type B1 periprosthetic fracture was diagnosed, indicating a need for reoperation. After extensive discussion with the patient and obtaining his informed consent, the revision with implantation of four cerclages took place the next day. Our patient has fully healed and is very satisfied with the result. The short-term result has been satisfactory. Our patient is able to walk without aids and is without symptoms on the left side. Treatment of the opposite side is planned.

A dual-energy X-ray absorptiometry (DEXA) to measure bone mineral density before surgery might be helpful and was not obtained in our case. Although DEXA is still the gold standard to measure bone mineral density, density can also be accurately determined on a pelvic CT scan [32].

All general limitations of a retrospective analysis apply to this study. Since this is a case report of only one patient, no control group exists and bone quality in the hip might vary in individual patients with MPS type IV. Additional limitations are the short time of follow-up (6 months) and the absence of further X-ray images, showing the healing process and osteointegration of the prothesis.

## 4. Conclusion

In conclusion, MPS type IVB is a rare autosomal recessive lysosomal storage disorder associated with highly specific skeletal deformities. Currently, no cure for Morquio's syndrome exists; therefore, supportive treatment to improve quality of life remains the central pillar of care. This case illustrates that the quality of life can be significantly increased by performing THA but should be approached with tact and sensitivity during implantation of the alloplastic material. Because of the age and life expectancy of the patient, an uncemented prosthesis was chosen. However, when there is poor bone quality, a cemented prosthesis should be considered.

## Data Availability

Original data can be received from the corresponding author upon reasonable request.

## Abbreviations

AC: Acetabular angle
DEXA: Dual-energy X-ray absorptiometry
DDH: Developmental dysplasia of the hip
GAG: Glycosaminoglycans
KS: Keratin sulfate
LCE: Lateral center-edge angle
MPS: Mucopolysaccharidosis
MRI: Magnetic resonance imaging
NAS: Numerical analog scale
ONFH: Osteonecrosis of the femoral head
ROM: Range of motion
THA: Total hip arthroplasty
VDRO: Varus derotation osteotomy.
